# Small cell lung cancer transformation and T790M mutation: complimentary roles in acquired resistance to kinase inhibitors in lung cancer

**DOI:** 10.1038/srep14447

**Published:** 2015-09-24

**Authors:** Kenichi Suda, Isao Murakami, Kazuko Sakai, Hiroshi Mizuuchi, Shigeki Shimizu, Katsuaki Sato, Kenji Tomizawa, Shuta Tomida, Yasushi Yatabe, Kazuto Nishio, Tetsuya Mitsudomi

**Affiliations:** 1 Division of Thoracic Surgery, Department of Surgery, Kinki University Faculty of Medicine, Osaka-Sayama, Japan; 2 Department of Surgery and Science, Graduate School of Medical Sciences, Kyushu University, Fukuoka, Japan; 3 Department of Respiratory Medicine, Higashi-Hiroshima Medical Center, Higashi-Hiroshima, Japan; 4 Department of Genome Biology, Kinki University Faculty of Medicine, Osaka-Sayama, Japan; 5 Division of Molecular Pathology, Department of Pathology, Hyogo College of Medicine, Nishinomiya, Japan; 6 Department of Pathology and Molecular Diagnostics, Aichi Cancer Center Hospital, Nagoya, Japan

## Abstract

Lung cancers often harbour a mutation in the epidermal growth factor receptor (*EGFR*) gene. Because proliferation and survival of lung cancers with *EGFR* mutation solely depend on aberrant signalling from the mutated EGFR, these tumours often show dramatic responses to EGFR tyrosine kinase inhibitors (TKIs). However, acquiring resistance to these drugs is almost inevitable, thus a better understanding of the underlying resistance mechanisms is critical. Small cell lung cancer (SCLC) transformation is a relatively rare acquired resistance mechanism that has lately attracted considerable attention. In the present study, through an in-depth analysis of multiple EGFR-TKI refractory lesions obtained from an autopsy case, we observed a complementary relationship between SCLC transformation and *EGFR* T790M secondary mutation (resistance mutation). We also identified analogies and differences in genetic aberration between a TKI-refractory lesion with SCLC transformation and one with *EGFR* T790M mutation. In particular, target sequencing revealed a *TP53* P151S mutation in all pre- and post-treatment lesions. *PTEN* M264I mutation was identified only in a TKI-refractory lesion with SCLC transformation, while *PIK3CA* and *RB1* mutations were identified only in pre-treatment primary tumour samples. These results provide the groundwork for understanding acquired resistance to EGFR-TKIs via SCLC transformation.

Lung cancers with epidermal growth factor receptor (*EGFR*) gene mutation account for ~40% of lung adenocarcinomas in East Asians and ~20% in Caucasians and African Americans^1^. Because proliferation and survival of lung cancers with *EGFR* mutation solely depend on aberrant signalling from the mutated EGFR, these tumours often show dramatic responses to EGFR tyrosine kinase inhibitors (TKIs)^2^. However, despite the initial response, emergence of acquired resistance to these drugs is almost inevitable, resulting in median progression-free survival ranging from 9.6–13.7 months^1^. Many acquired resistance mechanisms and candidates have been reported so far, such as *EGFR* T790M secondary mutation, *MET* gene amplification, *ERBB2* gene amplification, overexpression of hepatocyte growth factor, downregulation of PTEN, transformation to small cell lung cancer (SCLC), and epithelial to mesenchymal transition^3^,^4^. Among these resistance mechanisms, relapsed tumours with *EGFR* T790M secondary mutation and those with SCLC transformation can be treated by “resistance mechanism-based” therapies, such as T790M-specific EGFR-TKIs in clinical trial settings^5^ or cytotoxic chemotherapy and radiation for SCLC^3^.

SCLC transformation is a relatively rare acquired resistance mechanism in lung cancers with *EGFR* gene mutation. Details of less than 30 patients have been reported in 11 papers so far, based on our literature search^3^,^6^,^7^,^8^,^9^,^10^,^11^,^12^,^13^,^14^,^15^. However, this acquired resistance mechanism has lately attracted considerable attention as SCLC transformation can be diagnosed by standard pathological examination, and SCLC-specific treatment often shows clinical benefit^3^.

In the near future, specific resistance mechanism-based therapies will become more common through the analysis of biopsied small samples or pleural effusion. However, many patients also harbour multiple EGFR-TKI-refractory tumours simultaneously at the time of tumour burden. Therefore, it is important to understand inter-tumour heterogeneity of acquired resistance mechanism(s) in a single patient after treatment failure of EGFR-TKIs.

## Results

### Patient and clinical course

**Figure 1:**
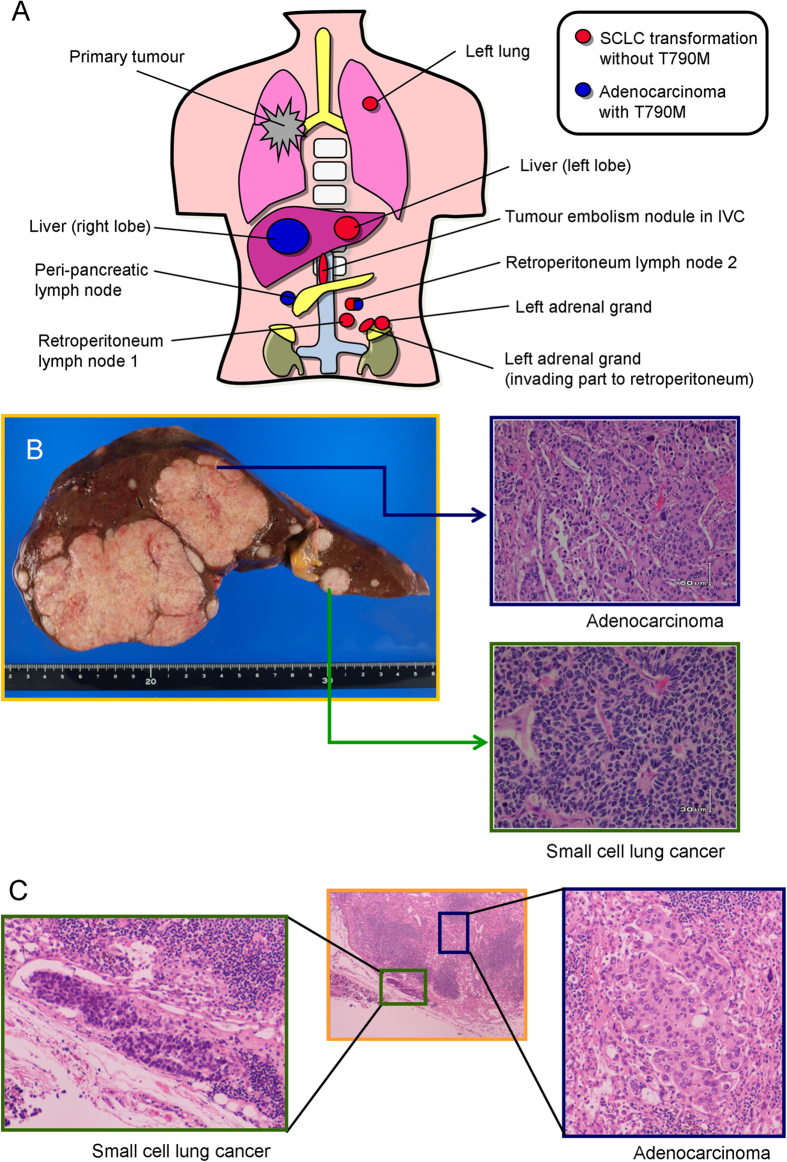
Anatomical and pathological examination of gefitinib-refractory metastatic lesions of the patient. (**A**) Schema of the metastatic lesions available. There were no viable tumour cells in the primary lung tumour. Red lesions indicate adenocarcinoma histology, and all adenocarcinoma lesions harboured the T790M mutation. Blue lesions indicate SCLC histology, and none of the SCLC lesions had the T790M mutation. One retroperitoneum lymph node possessed both the adenocarcinoma component with a T790M mutation and the SCLC component, independently. (**B**) Macroscopically, there were two types of tumours in the liver. Lesions in the right lobe consisted of adenocarcinoma histology. Lesions in the left lobe showed SCLC histology. (**C**) Detail of the retroperitoneum lymph node that possessed both the adenocarcinoma and SCLC components is shown.

Among 16 autopsy cases that met the clinical definition of acquired resistance to EGFR-TKIs^16^, one patient developed SCLC transformation. The patient was a 76-year-old female at diagnosis of lung cancer, without smoking history. She was initially treated with platinum-doublet chemotherapy with concurrent radiation for her clinical stage IIIB non-small cell lung cancer (NSCLC). Fifteen months later, she experienced tumour relapse with multiple lung metastases. She was treated with gefitinib monotherapy because her initial trans-bronchial lung biopsy sample harboured an *EGFR* exon 19 deletion mutation (E746_A750 del). Although partial response was obtained, acquired resistance developed 5 months later. Gefitinib was continued for an additional 3 months until her death with palliative radiation therapy for her cervical lymph node metastases. Ten tumour samples of the gefitinib-refractory metastatic lesions were obtained at the autopsy (Fig. 1A,B).

### Reciprocal relationship between SCLC transformation and EGFR T790M mutation

There were nine EGFR-TKI-refractory tumour lesions available, while there were no viable tumour cells in the primary lung tumour. Histologically, the nine metastatic lesions consisted of six SCLCs, two adenocarcinomas, and one retroperitoneum lymph node that included each histology independently (Fig. 1C). Genomic DNA was extracted separately from the adenocarcinoma and SCLC components from the retroperitoneum lymph node by a pathologist (S.S.).

All metastatic lesions harboured an *EGFR* exon 19 deletion mutation. Additionally, all lesions with adenocarcinoma histology harboured an *EGFR* T790M mutation, while none of the SCLC components possessed this secondary mutation in the *EGFR* gene (Fig. 1A). None of the lesions showed gain of the *MET* gene copy number.

### Mutational spectrum of pre-treatment and EGFR-TKI-refractory lesions

Next, we compared the mutational spectrum of pre-treatment primary tumours and two EGFR-TKI-refractory liver metastases, one with adenocarcinoma and the other with SCLC. As shown in Table 1, all pre-treatment tumours and both the post-treatment lesions harboured the *EGFR* E746_A750 deletion mutation and *TP53* P151S mutation. Presence of the *EGFR* T790M secondary mutation was confirmed in the post-treatment liver tumour with adenocarcinoma histology. The liver metastatic tumour with SCLC transformation harboured a *PTEN* M264I mutation (c. 792G > A), which was not detected in the tumour with adenocarcinoma histology. The pre-treatment lung tumour harboured *PIK3CA* and *RB1* mutations; however, these mutations were not detected in the relapsed liver tumours. All mutations identified in liver tumours were confirmed by a commissioned analysis performed by Life Technologies (Pervenio Lung NGS Panel) that evaluated 25 key genes associated with NSCLC. Presence of the *PTEN* M264I mutation was analysed by direct sequencing method in TKI-refractory tumours other than liver metastases (metastases in left lung, peri-pancreatic lymph node, retroperitoneum lymph node 1, tumour embolism nodule in IVC, left adrenal grand, and left adrenal grand invading part to retroperitoneum); however, none harboured this mutation.

## Discussion

As the first step in deciding a treatment strategy for a lung cancer patient, clinicians classify lung cancers into SCLCs or NSCLCs, because these two subclasses differ significantly in their oncological features and responses to radiation or chemotherapy. Therefore, it is of interest that lung adenocarcinomas, one of the subclasses of NSCLC, transform into SCLC in response to EGFR-TKI treatment. One possible explanation is that the SCLC component exists prior to the EGFR-TKI therapy (combined SCLC), and SCLC emerges from regression of the adenocarcinoma in response to EGFR-TKIs. However, it is plausible that SCLC and adenocarcinoma components both arose from the same origin, because in our case each histologic portion shared the same mutations in *EGFR* and *TP53*. In previously reported cases that experienced acquired resistance to EGFR-TKI via SCLC transformation^3^,^6^,^7^,^8^,^9^,^10^,^11^,^12^,^13^,^14^,^15^, all SCLC components also harboured the same activating *EGFR* mutation as the adenocarcinoma component.

The molecular mechanisms underlying SCLC transformation remain unclear. In this study, we identified the *PTEN* M264I mutation in a gefitinib-refractory liver tumour with SCLC transformation but not in a tumour with an adenocarcinoma component. The *PTEN* M264R mutation was previously reported in a patient with malignant melanoma, according to the COSMIC database. However, this *PTEN* M264I mutation is novel. The PTEN tumour suppressor gene negatively regulates the PI3K-AKT anti-apoptotic and proliferation pathway^17^. In lung adenocarcinoma, mutation of the *PTEN* gene is rare^18^,^19^,^20^, therefore the *PTEN* M264I somatic mutation in the liver tumour with SCLC transformation may play a role. A previous report by Sequist *et al*. reported SCLC transformation that developed together with *PIK3CA* mutation. These observations suggest the importance of the PI3K-AKT pathway in SCLC transformation. However, activation of this pathway is not enough, because an activating mutation of the *PIK3CA* gene is present in approximately 5% of NSCLCs^21^. Further analyses are necessary to uncover molecular mechanisms underlying SCLC transformation. Notably, there is a remarkable shared characteristic of patients who developed SCLC transformation after EGFR-TKI treatment failure; most of the patients were female with non-smoking history. Sex differences such as hormone levels or hormonal receptors may provide a clue for uncovering the process of SCLC transformation.

In this study, we observed a reciprocal relationship between SCLC transformation and *EGFR* T790M mutation. So far, only five patients have been reported who developed SCLC transformation in response to EGFR-TKI therapy and who had the data for multiple metastatic lesions (Table 2). In cases 1–3 shown in Table 2, all metastatic lesions, except for brain metastasis in case 3, showed SCLC transformation without evidence of *EGFR* T790M mutation. The brain metastasis of case 3 was adenocarcinoma without T790M mutation or *MET* amplification. It is possible that the brain metastasis did not acquire resistance to EGFR-TKI, but survived because of the low concentration of EGFR-TKI owing to the blood–brain barrier. However, in cases 4 and 5, some lesions harboured T790M mutation, while others showed SCLC transformation, suggesting a reciprocal relationship, as in our case. From these cases, it can be concluded that SCLC transformation and *EGFR* T790M mutation sometimes occur in a reciprocal manner in a single patient upon acquisition of resistance to EGFR-TKIs. Additionally, lesions with SCLC transformation may occupy a main part of the EGFR-TKI-refractory metastatic lesions, probably because of aggressiveness of SCLC compared with adenocarcinoma. These results suggest the appropriateness of using cytotoxic chemotherapy for SCLC, if one biopsied sample after EGFR-TKI treatment failure revealed SCLC transformation.

Several reports have analysed tumour heterogeneity in acquired resistance mechanisms to EGFR-TKI in lung cancers. In our previous analysis, two patients out of six showed heterogeneity of acquired resistance molecular mechanisms, showing a reciprocal relationship between T790M mutation and *MET* gene amplification^22^. In addition, Scher *et al*. reported a case that developed EGFR-TKI-refractory brain metastasis with T790M mutation and a lung lesion that showed squamous histologic transformation without T790M mutation^23^. These reports, together with the current study that observed a reciprocal relationship between SCLC transformation and *EGFR* T790M mutation, indicate that we have to keep the possibility of inter-tumour heterogeneity of resistance mechanisms in mind when treating patients using resistance mechanism-specific therapies based on the molecular analysis of biopsied small samples. Interestingly, a recent study that analysed acquired resistance mechanisms to T790M-specific EGFR-TKI identified SCLC transformation (and loss of T790M mutation)^24^.

Through the clinical application of EGFR-TKIs, the prognosis of lung cancer patients with *EGFR* mutation has dramatically improved^25^. Future studies should focus on better understanding the mechanisms of resistance and strategies to overcome acquired resistance to these drugs.

## Materials and Methods

### Patient and clinical course

Details of the patient and clinical course are described in the Results section. This study was approved by the Ethics Committee of Kinki University Faculty of Medicine and by the Ethics Committee of Higashi-Hiroshima Medical Center. Written informed consent for use of these tumour specimens was obtained from the patient’s legal guardians, and all samples were collected in accordance with the ethical guidelines.

### Analyses for EGFR T790M mutation and MET gene copy numbers

Extracted genomic DNA was used for the molecular analyses. The mutational status at codon 790 of the *EGFR* gene was analysed using the Cycleave PCR method, as described previously^26^. *MET* gene copy number was measured by quantitative real-time PCR using the SYBR Green Method (Power SYBR Green PCR Master Mix; Qiagen, Venlo, Netherlands) with a StepOnePlus system (Life Technologies, Foster City, CA, USA), as described previously^26^.

### Target sequencing analysis

Target sequencing was used to compare the mutational spectrum between two metastatic liver tumours (one with SCLC and the other with adenocarcinoma) and the pre-treatment biopsy sample of the primary tumour, as described previously^27^. Briefly, 20 ng of gDNA was used for the multiplex PCR amplification using the Ion AmpliSeq Library Kit and the Ion AmpliSeq Cancer Hotspot Panel v2 (Life Technologies) according to the manufacturer’s instructions. The Ion Xpress Barcode Adapters (Life Technologies) were ligated into the PCR products and reactions were purified with Agencourt AMPure XP beads (Beckman Coulter, Brea, CA, USA). Purified libraries were pooled and sequenced on an Ion Torrent PGM device (Life Technologies) using the Ion PGM 200 Sequencing Kit v2 and the Ion 318 v2 Chip Kit. DNA sequencing data were accessed through the Torrent Suite v. 4.2 software program.

### Direct sequencing analysis

The presence of the *PTEN* M264I mutation was analysed by direct sequencing method. Briefly, polymerase chain reaction (PCR) analysis of extracted genomic DNA (100 ng) was carried out using AmpliTaq Gold (Life Technologies). PCR primers were as follows: 5′-CCGTTACCTGTGTGTGGTGATA-3′ (forward) and 5′-ATGCCAGAGTAAGCAAAACACC-3′ (reverse). PCR products were diluted and cycle sequenced using the BigDye Terminator Cycle Sequencing Kit (Life Technologies), and sequencing reaction products were separated electrophoretically on an ABI PRISM 3130 apparatus.

## Additional Information

**How to cite this article**: Suda, K. *et al*. Small cell lung cancer transformation and T790M mutation: complimentary roles in acquired resistance to kinase inhibitors in lung cancer. *Sci. Rep*. **5**, 14447; doi: 10.1038/srep14447 (2015).

## Figures and Tables

**Table 1:** Summary of primary lesion and metastatic tumours.

Status^1^	Organ^2^	Histology	EGFR	TP53^4^	PTEN^4^	PIK3CA^4^	RB1^4^
Pre.	Right lung	Non-small cell lung cancer	E746_A750del	P151S	wt	E545K	E458^*^
Post.	Liver	Small cell lung cancer	E746_A750del	P151S	M264I	wt	wt
Post.	Liver	Adenocarcinoma	E746_A750del+T790M	P151S	wt	wt	wt
Post.	Left lung	Small cell lung cancer	E746_A750del	n.d.	wt	n.d.	n.d.
Post.	Peri-panc.	Adenocarcinoma	E746_A750del+T790M	n.d.	wt	n.d.	n.d.
Post.	Ret-LN 1	Small cell lung cancer	E746_A750del	n.d.	wt	n.d.	n.d.
Post.	Ret-LN 2^3^	Small cell lung cancer	E746_A750del	n.d.	n.d.	n.d.	n.d.
		Adenocarcinoma	E746_A750del+T790M	n.d.	n.d.	n.d.	n.d.
Post	IVC	Small cell lung cancer	E746_A750del	n.d.	wt	n.d.	n.d.
Post.	Left AG	Small cell lung cancer	E746_A750del	n.d.	wt	n.d.	n.d.
Post.	Left AG-inv.	Small cell lung cancer	E746_A750del	n.d.	wt	n.d.	n.d.

^1^Sample obtained from biopsy prior to gefitinib treatment (Pre.) or autopsy samples after gefitinib treatment failure (Post.).

^2^Peri-panc., Ret-LN, IVC, AG, and AG-inv. indicate peri-pancreatic lymph node, retroperitoneum lymph node, tumour embolism in inferior vena cava, adrenal grand, and adrenal grand invading part to retroperitoneum, respectively.

^3^Retroperitoneum lymph node 2 included each histology independently.

^4^n.d. indicates not done.

**Table 2:** Summary of patients from the literature who have data for multiple TKI-refractory lesions with SCLC transformation.

Case	Author	Age^1^	Sex	Mut.^2^	TKI^3^	Details of the patient	Ref.
1	Zakowski, *et al*.	45	F	19 del	erlotinib → gefitinib	Examined lesions from five organ sites all showed SCLC with no mutations in exons 18–24 of EGFR or exon 2 of KRAS.	6
2	Morinaga, *et al*.	46	F	19 del	gefitinib	Resected brain tumour and biopsied lung lesion both showed SCLC.	7
3	Sequist, *et al*.	67	F	L858R	erlotinib	Metastatic lesions in the lung, thoracic lymph nodes, liver, and nodules along the diaphragm all showed SCLC without T790M or MET amp. Brain metastasis retained adenocarcinoma without T790M or MET amp.	3
4	Niederst, *et al*.	56	F	L858R	erlotinib	Metastatic lesions in the lung and liver showed SCLC transformation with PIK3CA E545K mutation (without T790M), while the diaphragm tumour showed adenocarcinoma with T790M (without PIK3CA mutation).	3,15
5	Fallet, *et al*.	44	F	19 del	erlotinib	Tumour stenosis of the upper-right lobe bronchus showed SCLC without T790M, while bronchial aspirate showed adenocarcinoma with T790M.	9
6	Present case	76	F	19 del	gefitinib	Most metastatic lesions (7/9) had the SCLC component. TKI-refractory lesions with adenocarcinoma histology, but not SCLC, only harboured T790M.	—

^1^Age at initial diagnosis of lung cancer.

^2^Type of EGFR-activating mutation (19 del: exon 19 deletion; or L858R point mutation).

^3^Type of EGFR tyrosine kinase inhibitor(s).
